# Trigeminal Nerve White Matter Fiber Abnormalities in Primary Trigeminal Neuralgia: A Diffusion Spectrum Imaging Study

**DOI:** 10.3389/fneur.2021.798969

**Published:** 2022-01-20

**Authors:** Si-ping Luo, Fan-fan Chen, Han-wen Zhang, Fan Lin, Guo-dong Huang, Yi Lei

**Affiliations:** ^1^College of Medicine, Shantou University, Shantou, China; ^2^Department of Radiology, Shenzhen Second People's Hospital, The First Affiliated Hospital of Shenzhen University, Health Science Center, Shenzhen, China; ^3^Department of Neurosurgery, Shenzhen Second People's Hospital, The First Affiliated Hospital of Shenzhen University, Health Science Center, Shenzhen, China

**Keywords:** trigeminal neuralgia, diffusion spectrum imaging, diffusion tensor imaging, fiber tractography, diffusion magnetic resonance image

## Abstract

**Objective:**

Diffusion spectrum imaging (DSI) was used to quantitatively study the changes in the trigeminal cistern segment in patients with trigeminal neuralgia (TN) and to further explore the value of acquiring DSI data from patients with TN.

**Methods:**

To achieve high-resolution fiber tracking, 60 patients with TN and 35 healthy controls (HCs) were scanned with conventional magnetic resonance imaging (MRI) and DSI. The patients and the members of the control group were compared within and between groups. The correlations between quantitative parameters of DSI and the visual analog scale (VAS), and symptom duration and responsible vessel types were analyzed.

**Results:**

Compared with unaffected side of patients in the TN group, the affected side showed significantly decreased quantitative anisotropy (QA) (*p* < 0.001), fractional anisotropy (FA) (*p* = 0.001), and general FA (GFA) (*p* < 0.001). The unaffected side exhibited significantly decreased QA (*p* + 0.001), FA (*p* = 0.001), and GFA (*p* < 0.001) and significantly increased axial diffusivity (AD) (*p* = 0.036) compared with the affected side of patients in the TN group and the average values of HCs. There were significantly decreased QA (*p* = 0.046) and FA (*p* = 0.008) between the unaffected side of patients and the average values of HCs. GFA can evidently distinguish arteries, veins, and features of unaffected side in TN patients.

**Conclusion:**

Using high-resolution fiber tracking technology, DSI can provide quantitative information that can be used to detect the integrity of trigeminal white matter in patients with TN and can improve the understanding of the disease mechanism.

## Introduction

Trigeminal neuralgia (TN) is recurrent, unilateral, transient, and electric pain in the trigeminal nerve distribution area. It has been reported that the annual incidence is about 4–29/10 million person-years worldwide (1). The prevalence rate in women was higher than that in men (F:M = 3:2). With disease aggravation, pain attacks become more frequent, affecting basic human functions, such as speaking, eating, drinking, or touching the face, resulting in low quality of life (1).

The etiology of primary TN is unknown. At present, the generally accepted theory is neurovascular compression (NVC). This theory suggests that microvascular compression leads to demyelination of trigeminal nerve roots, causing TN. These vessels that compress the trigeminal nerve are called responsible vessels. Microvascular decompression (MVD), a minimally invasive interventional technique, is designed based on this etiological explanation and has been recognized as the most effective method for the treatment of TN (2, 3). MVD can identify the painful nerve and effectively isolate the responsible vessels that compress the trigeminal nerve root and brainstem to relieve compression and, repair nerve pain under the operating microscope and eliminate the source of trigeminal nerve pain (4).

As a non-invasive magnetic resonance imaging (MRI) technique to evaluate the integrity of white matter, diffusion tensor imaging (DTI) can indirectly reflect the integrity of nerve fiber bundles by measuring the diffusion movement of water molecules (5), thus providing new insights into the etiology of TN. Previous studies (6, 7) have shown that in patients with TN, DTI parameters such as fractional anisotropy (FA) decreased significantly and axial diffusivity (AD), radial diffusivity (RD), and mean diffusivity (MD) increased significantly. However, some studies (8, 9) argued different or even opposite opinions of the changes of DTI metrics values between affected and unaffected sides in patients and controls. In addition, the correlation between the degree of FA decrease and the degree of pain, symptom duration, and types of responsible vessels are controversial (8, 10, 11). Moreover, DTI needs larger sample sizes for standardization and the spatial resolution limits the further use of DTI in patients with TN.

Diffusion spectrum imaging (DSI) generalizes DTI by acquiring more directions in q-space, either by high-angular resolution diffusion imaging shells, a cube on a Cartesian grid, or Q-ball imaging, which has been used to non-invasively detect the complex structure of white matter bundles and fiber bundles in human brain (12). DSI reconstructs fiber bundles with higher resolution than traditional DTI and has been shown to accurately display crossing, winding, interruption, and small fibers (13, 14). DSI parameters include DTI parameters: FA, RD, MD, and AD as well as unique parameters: quantitative anisotropy (QA), general FA (GFA), restricted diffusion imaging (RDI), and isotropic diffusion component (ISO). The GFA value, which represents the direction consistency of water molecule diffusion and reflects the integrity of axon or myelin more accurately and sensitively than FA value, is the main quantitative parameter of DSI (15, 16). Generalized q-sampling imaging (GQI) (17) is one of the commonly used DSI data reconstruction models for calculating QA and ISO. RDI (18) is a model-free method to quantify the density of restricted diffusion given a diffusion displacement range. DSI has been used to study some mental diseases, such as autism (19) and schizophrenia (20), as well as neurodegenerative diseases, such as Alzheimer's disease (21) and multiple sclerosis (22). Recently, DSI has been increasingly used to study the brain nerve structure (23).

In this study, we used DSI technology to study and compare the changes in cistern segments between the affected side and the unaffected side of patients with TN, analyze the difference of DSI parameters between patients with TN and healthy controls (HCs), and the relationship between the degree of difference value and symptom duration, the degree of pain, and responsible vessels. We hypothesized that some of the identified white matter abnormalities exist in TN patients and would correlate with the degree of pain, symptom duration, and responsible vessel types, so as to further explore the value of utilizing DSI technology in patients with TN.

## Materials and Methods

### Participants

This study collected patients with TN diagnosed in the Shenzhen Second People's Hospital from November 2019 to August 2021. They all underwent MRI and excluded: (1) TN secondary to other neurological conditions such as tumor growth or multiple sclerosis; (2) There are other known intracranial lesions including obvious trauma; (3) Previous history of craniocerebral surgery; and (4) Incomplete image data. After screening, a total of 60 patients were included in this study. By posting advertisements in several health management centers, 35 years of age and sex-matched participants without pain and any neurological diseases served as HCs. The protocol was approved by the Hospital Bioethics Committee. All the participants received a written informed consent prior to registration.

### Magnetic Resonance Imaging Acquisition

Scanning was performed on a 3.0-T MRI system (Prisma, Siemens) using a 20-channel head coil. Comfortable and tight foam padding was used to limit head movement. All the patients were treated with three-dimensional time-of-flight magnetic resonance angiography (3D-TOF-MRA) and T2_SPC_TRA_ISO sequence before operation to check the relationship between blood vessels and nerves. The relevant parameters for the T2_ SPC sequence were repetition time/echo time (TR/TE) = 1,000 ms/125 ms, field of view (FOV) = 82 mm × 160 mm, matrix = 320 × 164, 2 average, slice thickness = 0.5 mm, flip angle 100°, and scanning time: 3 min 46 s; 3D-TOF sequence: TR/TE = 20 ms/3.69 ms, FOV = 191 × 200 mm, matrix = 384 × 331, 1 average, slice thickness = 0.5 mm, flip angle 18°, and scanning time: 4 min 49 s.

Data were collected using MRI sequences including T1-weighted magnetization-prepared rapid acquisition gradient-echo (MPRAGE) for better anatomic reference and DSI using pulsed gradient twice-refocused spin-echo echo-planar imaging (EPI) sequences. T1-weighted sagittal images were obtained with 3D fast-field echo imaging. The following acquisition parameters were employed: repetition time, 2,300 ms; echo time, 3.55 ms; flip angle, 8°; slice thickness, 0.9 mm; number of sections, 192; FOV, 240 × 240 mm; matrix, 256 × 256; and voxel size, 0.9 × 0.9 mm. The acquisition parameters for DSI were as follows: repetition time, 6,300 ms; echo time, 71 ms; slice thickness, 2.2 mm; number of sections in the transverse plane, 60; FOV, 220 × 100 mm; matrix, 220 × 100; and voxel size, 2.2 × 2.2 × 2.2 mm. A total of 128 directions with the maximum diffusion sensitivity bmax = 3,000 s/mm^2^ were sampled on the grid points in the 3D q-space. The grid sampling scheme sampled the diffusion encoding space using a given grid, which consisted of 19 *b*-values of *b* = 200, 350, 400, 550, 750, 950, 1,100, 1,150, 1,500, 1,700, 1,850, 1,900, 2,050, 2,250, 2,400, 2,450, 2,600, 2,650, 3,000 s/mm^2^ along 3, 2, 4, 4, 3, 12, 8, 4, 6, 15, 8, 4, 12, 4, 2, 10, 22, 2, and 3 directions, respectively.

### Clinical Data

The clinical data of all the patients with TN including sex, age, symptom duration, and pain laterality (right or left pain) were obtained from medical records. The main results of pain intensity were evaluated by using the visual analog scale (VAS) (0: no pain; 10: the most serious possible pain). The pain perception of patients with TN was measured by a senior pain physician.

The judgment of the responsible vessel types shall be diagnosed by the surgeon doctors and radiologists. For patients undergoing MVD surgery, the type of responsible vessel can be obtained by querying the operation records. If MVD surgery is not performed or the operation records are unclear, two senior radiologists will diagnose the type of responsible vessel through pre-operative MRI examination. The types of responsible vessels are divided into artery and vein.

### Tract Analysis

To analyze the microstructure integrity of white matter fiber bundles, we used the DSI Studio software (http://dsi-studio.Labsolver.org/) to reconstruct the fiber bundles of all the participants. The diffusion image was imported into the DSI Studio software, brain mask was set, and generalized q-sampling imaging (GQI) with diffusion sampling length ratio of 1.25 was used to reconstruct the model (17). Restricted diffusion was quantified using restricted diffusion imaging. The region of interest (ROI) is placed in the QA diagram, the volume sizes of the ROIs were 1.3 × 103 mm^3^-2.0 × 103 mm^3^ within the cisternal segment of the trigeminal nerve (24), and the trigeminal nerve can be seen in the brainstem area of bilateral trigeminal nerves. A deterministic fiber tracking algorithm is used to reconstruct the trigeminal nerve tracts (25). The quantitative anisotropy threshold was 0.20 ± 0.25 (subject dependent), the angular threshold was 60°, the step size was 1.2 mm, the smoothness was 0.80, the minimum length was 10 mm, and the maximum length was 200 mm. A total of 5,000 were calculated. Finally, the values of average GFA, QA, RDI, and ISO as well as the basic diffusion coefficients such as FA, MD, AD, and RD are calculated. Two radiologists with more than 10 years of neuroradiology experience tracked the seeds and targets, respectively, and obtained the average values of the above parameters.

### Difference of Parameters

The average value of each parameter for HCs was obtained from the average value of the left and right sides of these participants. The difference value of each parameter = average parameter value of HCs—parameter value of the affected side. The difference scores of each parameter = (average parameter value of HCs)—(parameter value of the affected side)/(average parameter value of HCs) (10). The difference value and difference scores of each parameter on the affected side and unaffected side of patients with TN can be obtained. The above parameter value, including the parameter value of TN affected side, unaffected side and HCs, difference value, and difference scores were statistically were used. Correlation analysis and logistic analysis were carried out to investigate relationships among the parameter value and VAS scores, symptom duration, and responsible vessel types.

All the patients were assigned to either the short-duration (symptom duration < 4 years) or the long-duration (symptom duration ≥ 4 years) groups and their affected side parameters were compared. The threshold was set to the mean symptom duration of all the trigeminal patients in our cohort. The statistics analysis was performed with *t*-test.

### Statistical Analysis

Continuous variables were summarized as means (±SD). For categorical variables, the percentages of patients in each category were calculated.

Statistical analyses were performed in the SPSS version 22.0 (IBM Corporation, Armonk, New York, USA). Differences between groups were assessed using the independent samples *t*-tests or the one-way ANOVA, whereas differences within groups were assessed using the paired-samples *t*-tests. Benjamini and Hochberg correction was used to correct the multiple comparisons of different groups. The Bonferroni correction was used to correct the multiple comparisons of intragroup comparison. Bivariate correlation and partial correlation analyses were used to identify the relationship between the DSI parameters that exhibited significant differences and clinical severity assessment. Normality of the data was assessed using the Shapiro–Wilk-test. For non-normally distributed variables, a non-parametric equivalent test was performed. If the data had unequal variances (determined by Levene's test for homogeneity of variances) and/or unequal sample sizes, Welch's *t*-test or Welch's ANOVA followed by Games–Howell *post-hoc* tests was performed. *p* < 0.05 was considered as statistically significant.

## Results

### Demographic and Disease Characteristics of Participants

Table 1 shows 60 patients diagnosed with primary TN (age 57.45 ± 8.78 years; range 25–80 years; 22 men and 38 women) and 35 HC (age 54.6 ± 5.2 years; range 45–65 years; 20 men and 15 women). Sex and age were not significantly different between the two groups of subjects. Affected regions were more often observed on the right side than the on left. Responsible vessels are mainly arteries (73.3%) and veins (26.7%).

**Table 1:** Demographic and disease characteristics of participants.

	**Patients (*n* = 60)**	**Controls (*n* = 35)**	***p*-value**
**Age (mean** **± SD, years)**	57.45 ± 8.786	54.6 ± 5.259	0.085^a^
**Sex (number, %)**			
Male	22 (36.7%)	20 (57.1%)	0.058^b^
Female	38 (63.3%)	15 (42.9%)	
**Disease duration (mean** **± SD, years)**	4.22 ± 4.04		
Short duration	35 (58.4%)	NA	NA
Long duration	25 (41.6%)		
**Pain side (number,%)**			
Right	38 (63.3%)	NA	NA
Left	22 (36.7%)	NA	
**VAS (mean** **± SD, score)**	8.45 ± 0.72	NA	NA
**Responsible vessels (number, %)**			
**Arteries**	44 (73.3%)		
Superior cerebellar artery (SCA)	30 (50%)	NA	
Anterior inferior cerebellar artery (AICA)	10 (16.7%)		NA
Basilar artery	4 (6.6%)		
**Veins**	16 (26.7%)	NA	

*VAS, visual analog scale; NA, not applicable*.

a *p-values were calculated with the two-tailed t-tests*.

b *p-value was obtained using the chi-squared test*.

### Diffusion Spectrum Imaging Analysis

**Figure 1:**
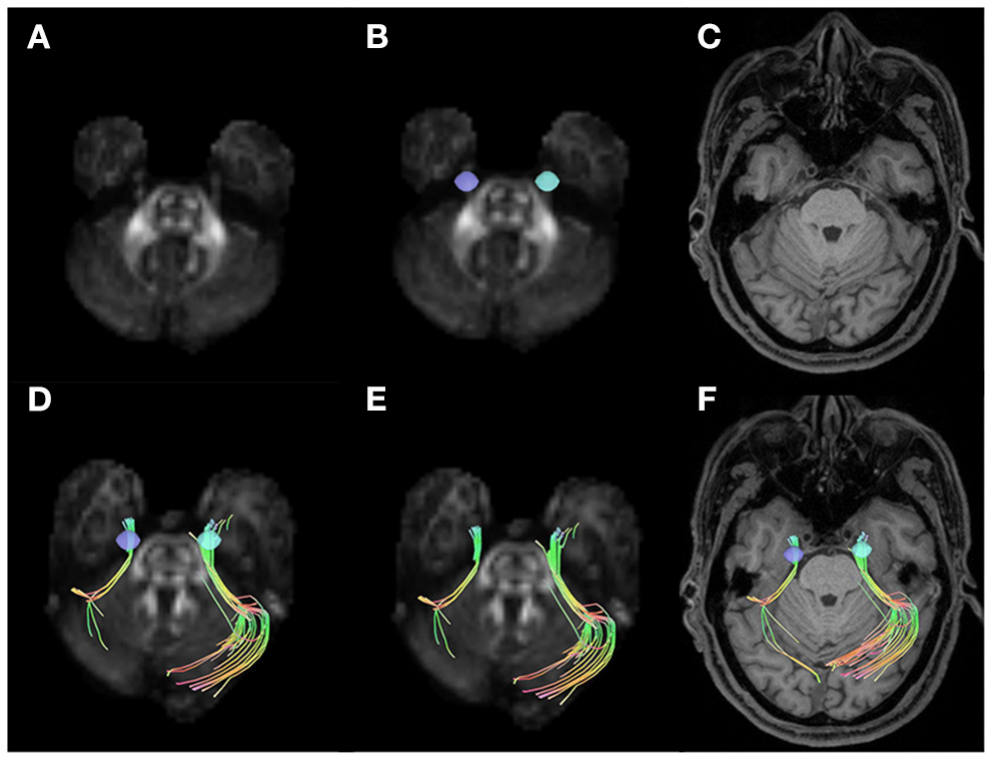
Examples of volumes of interest, trigeminal nerve tracking, and trigeminal nerve reconstruction. **(A)** Shows the quantitative anisotropy (QA) diagram. **(B)** Shows the seeds placed on two sides of the cistern segments in trigeminal nerve (purple and blue). **(C)** Shows the T1-weighted image (T1WI) structural MRIs. The trigeminal nerve tractography for right side affected patients with trigeminal neuralgia (TN) in QA diagram **(D,E)** and T1WI MRI **(F)**.

In all the participants, we successfully completed high-definition fiber tractography of the trigeminal nerve in cistern segments and an instance of a patient's tractography is displayed in Figure 1. Two radiologists measured and calculated the parameters separately and the intraclass correlation coefficients (ICCs) of these measurements were ICC_QA_ = 0.87, ICC_ISO_ = 0.96, ICC_MD_ = 0.93, ICC_AD_= 0.95, ICC_FA_ = 0.86, ICC_RD_ = 0.92, and ICC_GFA_ = 0.83. The parameters were averaged and analyzed.

After fiber tracking, DSI parameters were extracted for intragroup comparison. There was no significant difference in any parameters between the left and right sides of HCs. Compared with the unaffected side of patients in the TN group, the affected side showed significantly decreased QA (*p* < 0.001), FA (*p* = 0.001), and GFA (*p* < 0.001), as shown in Table 2.

**Table 2:** Compared with the sides of the affected and unaffected patients and the healthy control group left and right.

	**Patients (*****n*** **=** **60)**		***p*-value**	**Healthy controls (*****n*** **=** **35)**		***p*-value**
	**Affected**	**Unaffected**		**Left**	**Right**	
QA	0.0665 ± 0.008	0.0837 ± 0.016	<0.001	0.0788 ± 0.013	0.0779 ± 0.011	0.7
FA	0.2678 ± 0.047	0.2764 ± 0.043	0.001	0.3159 ± 0.1159	0.3023 ± 0.0487	0.425
GFA	0.0627 ± 0.010	0.0713 ± 0.007	<0.001	0.0695 ± 0.0058	0.0700 ± 0.0064	0.744

*QA, quantitative anisotropy; FA, fractional anisotropy; GFA, general FA*.

*p-values were calculated with the paired-samples t-tests*.

**Figure 2:**
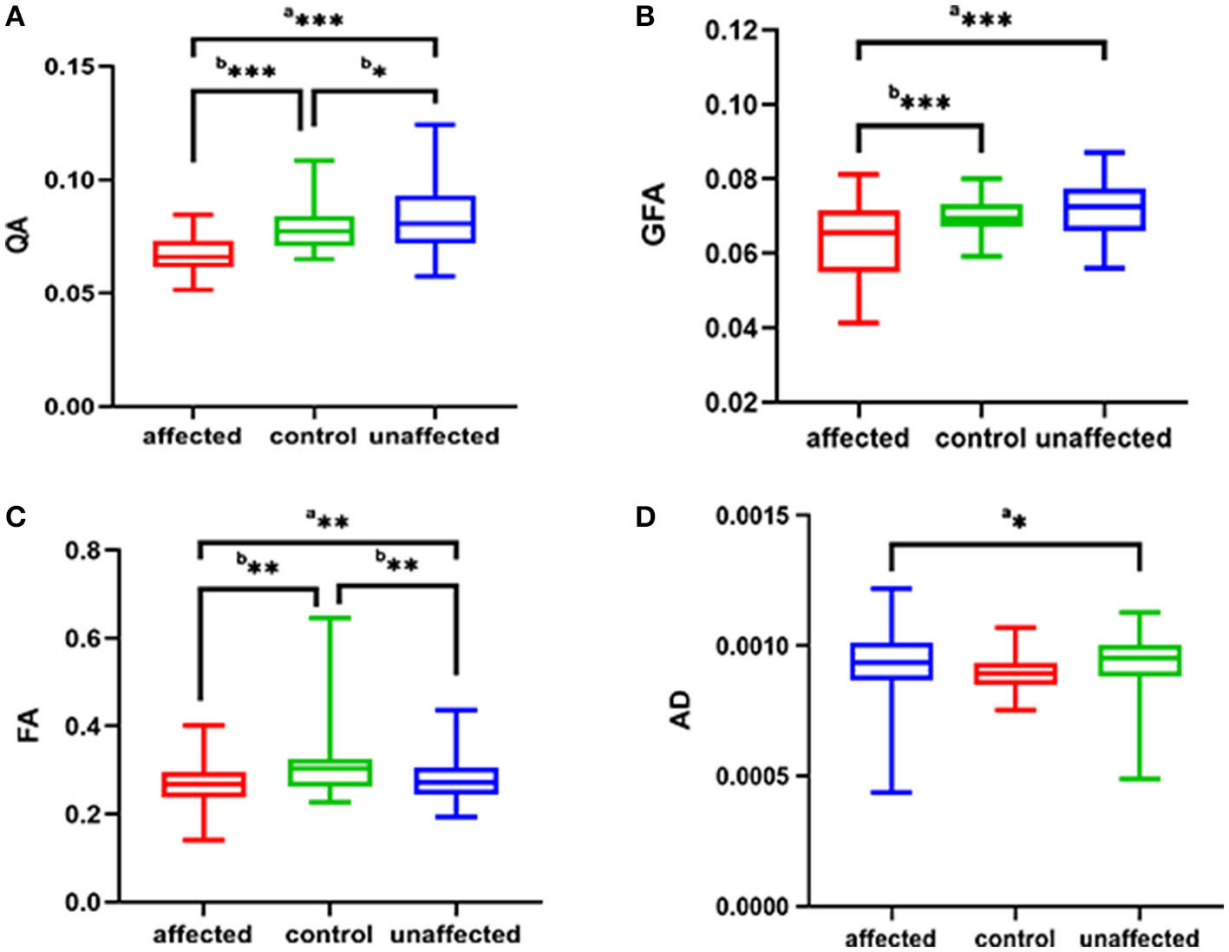
The **(A–D)** respectively shows QA, GFA, FA, AD of the affected side and unaffected side of patient with TN were compared with those average of the HC group. ^a^*p* was calculated with the paired-samples *t*-tests, ^b^*p* was calculated with the independent samples *t*-tests (***p < 0.001; **p < 0.01; *p < 0.05). QA, quantitative anisotropy; GFA, generalized FA; FA, fractional anisotropy; AD, axial diffusivity; HC, healthy control.

The parameters of the patient's affected side and unaffected side were compared with the average parameter values of the HCs. In the affected side of patients in the TN group, significantly decreased QA (*p* < 0.001), FA (*p* = 0.001), and GFA (*p* < 0.001) and significantly increased AD (*p* = 0.036) were noted compared with the average values of HCs. In addition, there were significantly decreased QA (*p* = 0.046) and FA (*p* = 0.008) between the patient's unaffected side and the average parameter values of HCs, as shown in Table 3 and Figure 2.

**Table 3:** Compared with patients and healthy controls.

	**Patient-unaffected**	**Healthy control**	***p*-value**	**Patient-affected**	**Healthy control**	***p*-value**
QA	0.08378 ± 0.0161	0.0783 ± 0.0099	0.046	0.0665 ± 0.0080	0.0783 ± 0.0099	<0.001
AD	0.00092 ± 0.0001	0.0008 ± 0.0001	0.059	0.00094 ± 0.0001	0.0008 ± 0.0001	0.036
FA	0.2764 ± 0.0435	0.3091 ± 0.0735	0.008	0.2678 ± 0.0469	0.3091 ± 0.0735	0.001
GFA	0.0713 ± 0.0071	0.0698 ± 0.0044	0.21	0.0627 ± 0.0102	0.0698 ± 0.0044	<0.001

*QA, quantitative anisotropy; AD, axial diffusivity; FA, fractional anisotropy; GFA, general FA*.

*p-values were calculated with independent sample t-test*.

### Difference of Parameters

**Figure 3:**
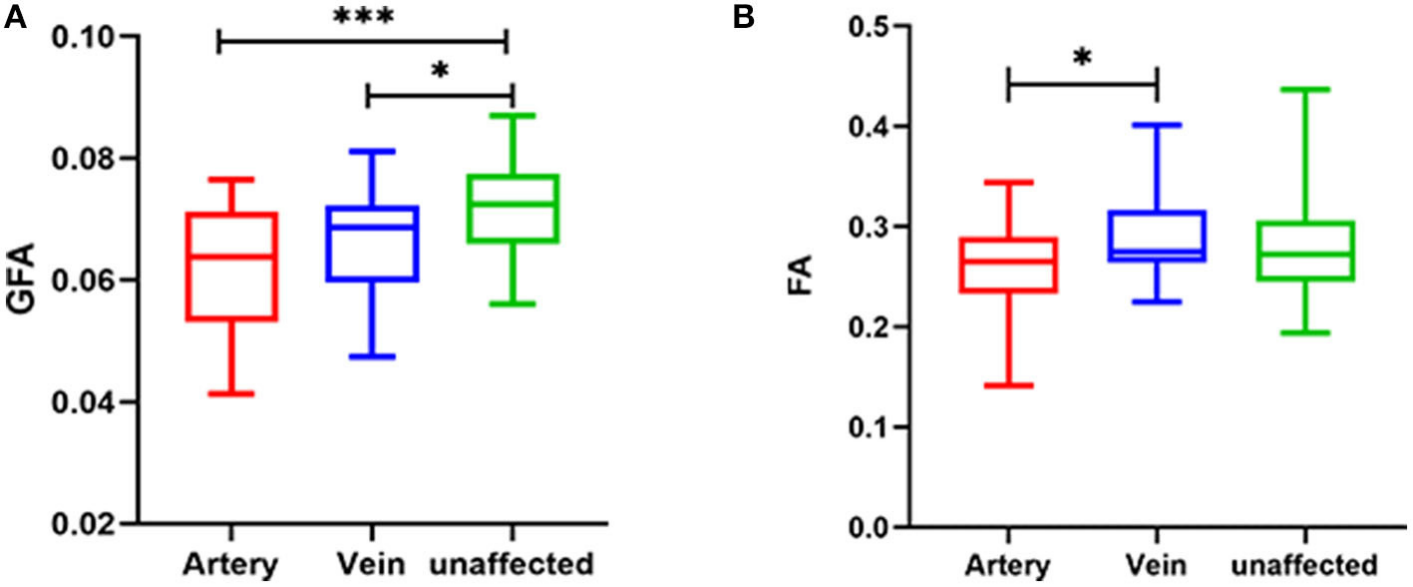
Relationship between responsible vessel types and GFA value **(A)** and FA value **(B)** in TN patients (***p < 0.001; *p < 0.05). GFA, generalized FA; FA, fractional anisotropy; DSI, diffusion spectrum imaging.

The significantly different parameters discussed above were used to calculate the parameter difference scores and correlation analysis and logistic analysis were carried out between the difference scores and VAS scores and symptom duration, and responsible vessel types. There was a correlation between TN patient's' VAS scores and symptom duration and there was no significant correlation between the difference scores of each parameter and VAS scores and between the difference scores of each parameter and symptom duration. In the TN group, FA (*p* = 0.028) was significantly decreased between arteryies and veins in responsible vessel types. GFA showed artery (*p* < 0.001), followed by veins (*p* = 0.017), in the affected side had the most significant decreases, compared with measurements of the unaffected side, while FA showed no significant difference between the affected artery and unaffected side, these findings are depicted in Table 4 and Figure 3.

**Table 4:** Compared responsible vessel types and the unaffected side in patients.

	**FA**	***p*-value**	**GFA**	***p*-value**
Arteries (*n* = 44)	0.25990 ± 0.0461	0.028	0.06150 ± 0.0105	0.12
Veins (*n* = 16)	0.28971 ± 0.0435		0.06617 ± 0.0089	
Arteries (*n* = 44)	0.25990 ± 0.0461	0.065	0.06150 ± 0.0105	<0.001
Unaffected side (*n* = 60)	0.27640 ± 0.0435		0.07133 ± 0.0071	
Unaffected side (*n* = 60)	0.28971 ± 0.0435	0.281	0.06612 ± 0.0089	0.017
Veins (*n* = 16)	0.27640 ± 0.0435		0.07133 ± 0.0071	

*FA, fractional anisotropy; GFA, general FA*.

*p-values were calculated with independent sample t-test*.

For affected side of the TN group, patients in the long-duration (25, 41.6%) group showed significantly decreased ISO (*p* = 0.021) and RDI (*p* = 0.021) compared with patients in the short-duration group (35, 58.4%), as shown in Table 5.

**Table 5:** Compare short-duration and long-duration groups.

	**Patients (*****n*** **=** **60)**		***p*-value**
	**Long duration (*n* = 25)**	**Short duration (*n* = 35)**	
ISO	0.2766 ± 0.0494	0.3097 ± 0.0553	0.021
RDI	0.1625 ± 0.0345	0.1850 ± 0.0371	0.021

*ISO, isotropic diffusion component; RDI, restricted diffusion imaging*.

*Short duration (symptom duration < 4 years), long duration (symptom duration ≥ 4 years)*.

*p-values were calculated with independent sample t-test*.

## Discussion

In this study, we explored the DSI parameters of trigeminal nerve in patients with TN and HCs. The results showed that QA, FA, and GFA on the affected side of individuals in the TN group decreased significantly, compared with the values on the unaffected side. On the affected side of individuals in the TN group, QA, FA, and GFA were significantly decreased and AD was significantly increased compared with the average values of HCs. There were significantly decreased QA and FA in the patient's unaffected side of patient than the average parameter values of HCs.

The etiology of primary TN is undefined. At present, the generally recognized theory is NVC. When the trigeminal nerve is compressed by blood vessels, the main cause of TN may be trigeminal nerve fiber bundle edema and demyelination (26). MVD well supports this theory. Routine MRI cannot suitably evaluate the integrity of trigeminal nerve white matter fiber bundle. Previous studies mostly used the traditional DTI method to study the white matter damage in patients with TN (27). Nevertheless, traditional DTI cannot accurately distinguish the cross fibers with limited angular resolution (28). In addition, DTI is affected by magnetic susceptibility artifact and partial volume average of complex fiber structure, which may lead to poor tracking (29).

In this study, we chose DSI instead of DTI to evaluate the damage of trigeminal nerve white matter fiber bundle. DSI has a longer acquisition time and provides more accurate quantitative details than DTI. DSI is a valuable tool for central nervous system imaging as it can be used to visualize the structural details of white matter bundles in multiple directions (14, 28–30). Similar to previous studies (6, 7), in our study, the FA value of trigeminal nerve on the affected side of TN patients was lower than that on the unaffected side and the decrease of FA value indicated that the integrity of white matter fiber bundle was damaged. However, in our study, AD, RD, and MD in the patient group did not increase significantly as previous studies. In this study, we found that FA, QA, and GFA values of the affected side of TN patients were significantly lower than those of the unaffected side and HCs. GFA was sensitive to axonal properties such as the integrity of the axonal membrane and myelin (31), whereas Yeh et al. (25) suggested that QA was more robust to free water effect and partial volume of crossing fibers. In addition, QA is an anisotropy index similar to FA, but it is calculated for each orientation-distribution function peak in each voxel. The decrease of QA and GFA can both reflect the damage to white matter, similar to FA (25). In our study, the decrease of QA and GFA values in TN patients might have indicated incompleteness of axon structure.

Consistent with previous studies (6), our study found that the QA and FA values of the unaffected side of TN patients decreased compared with those of HCs. QA and FA decreased, indicating that the integrity of trigeminal nerve fibers on the unaffected side of TN patients was damaged. Therefore, there may be microstructural abnormalities in the trigeminal nervous system in individuals the TN group. TN patients may be sensitive to chronic pain due to pre-existing white matter abnormalities, which may explain why some people do not develop TN even if they have NVC. Because of the previous abnormalities of trigeminal nerve (such as high AD value and/or MD, RD value, and low FA value) (32), vascular compression is enough to cause TN, and there may be individual differences in susceptibility to chronic pain (6).

In our study, there were more patients with right TN than left TN, which was consistent with the results of previous studies (8, 26). The main responsible vessel type is the artery, especially the superior cerebellar artery (SCA), followed by the anterior inferior cerebellar artery (AICA) and the basilar artery, which is also consistent with the previous study (8, 26).

In the study of correlation with clinical symptoms, previous studies found that the decline rate of FA in DTI was significantly correlated with VAS scores, symptom duration, and responsible vessel types (11). Other studies showed that the decline of FA was negatively correlated with VAS and symptom duration (11). However, in this study, the difference scores of DSI parameters were not significantly correlated with VAS, symptom duration, and responsible vessel types. However, we found that artery GFA had the most significant decrease, followed by veins, compared with the unaffected side of individuals in the TN group. Previous studies have shown that trigeminal artery compression results in more obvious pain symptoms and more serious nerve injury than venous compression because the pulsation of the artery is better than that of the vein. Moreover, the calculation of GFA is based on the orientation distribution function (ODF), which accurately and sensitively reflects the integrity of axon or myelin sheath than FA value and is very sensitive to axon characteristics such as the integrity of axon membrane and myelin sheath (11). Regardless of the presence of artery or vein compression, the white matter fiber bundle of trigeminal nerve is damaged. The injury in fiber bundle might be minimally difference, but the edema caused by compression may be significantly different. Hence, FA might be showed different, while GFA is not divided. It might explain why GFA can evidently distinguish between arteries, veins, and the unaffected side in TN patients while FA not.

In our understanding, the longer the onset of pain symptoms takes, the longer the trigeminal nerve and vascular compression is. Then, damage to trigeminal nerve microstructure is more serious in patients with long symptom duration. However, our study showed that there was no significant difference in QA, GFA, and FA between the patients with long and short symptom duration. Previous animal studies (33) have proved that the supposed pulsatile nature of pathological vascular contact results in a process of demyelination and remyelination of the nerve root, rather than progressive demyelination. This process, together with the altered pain management threshold, is considered to be the cause of the typical paroxysmal rather than persistent pain syndrome in patients with TN. Based on this result, it can be assumed that the changes of parameters reflect the alternating process of demyelination and remyelination, rather than linear progressive demyelination.

Furthermore, our results showed that patients with long symptom duration had ISO and RDI decreased compared with that of patients with short symptom duration. The ISO is the minimum distribution value of an ODF. It represents background isotropic diffusion contributed from cerebrospinal fluid (CSF) or non-directional restricted diffusion (34). To separate restricted ISO from non-restricted ISO, a spectrum of RDI measures estimating restricted or non-restricted diffusion was used (18). We suggested that it was possibly caused by long-term microvascular compression, so that the intercellular space was smaller and the isotropic diffusion of water molecules was restricted. This finding may support NVC theory.

We acknowledge that our study has some limitations. First, there will be some differences in the results of manual tracking settings, including the position and size of ROIs, and the anisotropy threshold. Secondly, the sample size of a single center is relatively small, which may have limited the difference between groups of some parameters, thus hindering the realization of statistical significance to a certain extent. There are some methodological considerations in previous research, such as DTI vs. DSI, REZ vs. cisternal segment of nerve for the ROI placement, averaging control nerves vs. comparing to just one side etc. These methodological differences may also be a source of discrepancy between the current study and previous literature. The current study was a cross-sectional study. The longitudinal study of TN patients in the future will help to evaluate the prognosis of patients before operation and verify the accuracy of this study. In the future, we hope to include more samples for research.

## Conclusion

We found that DSI parameters such as QA, GFA, FA decreased, and AD increased in TN patients. Moreover, GFA can evidently distinguish between arteries, veins, and the unaffected side in patients with TN. This findings reflects the change of trigeminal nerve microstructure and fiber integrity, which helps us to better understand the mechanism of disease.

## Data Availability Statement

The raw data supporting the conclusions of this article will be made available by the authors, without undue reservation.

## Ethics Statement

The studies involving human participants were reviewed and approved by Research Ethics Committee of the First Affiliated Hospital of Shenzhen University. The patients/participants provided their written informed consent to participate in this study.

## Author Contributions

S-pL, F-fC, FL, G-dH, and YL: manuscript editing. S-pL, F-fC, and FL: study conception/design, data analysis/interpretation and manuscript drafting, or revision for important intellectual content. S-pL and FL: literature research and data analysis. S-pL, F-fC, and H-wZ: data acquisition or clinical studies. All authors approval of final version of submitted manuscript and agrees to ensure any questions related to the work are appropriately resolved.

## Funding

This study is supported by a grant from Basic Plan Program of Shenzhen, China (No. JCYJ20180228163333734).

## Conflict of Interest

The authors declare that the research was conducted in the absence of any commercial or financial relationships that could be construed as a potential conflict of interest.

## Publisher's Note

All claims expressed in this article are solely those of the authors and do not necessarily represent those of their affiliated organizations, or those of the publisher, the editors and the reviewers. Any product that may be evaluated in this article, or claim that may be made by its manufacturer, is not guaranteed or endorsed by the publisher.

## References

[1] Lambru G Zakrzewska J Matharu M. Trigeminal neuralgia: a practical guide. Pract Neurol. (2021) 21:392–402. 10.1136/practneurol-2020-002782 34108244 PMC8461413

[2] Sindou M Brinzeu A. Topography of the pain in classical trigeminal neuralgia: insights into somatotopic organization. Brain. (2020) 143:531–40. 10.1093/brain/awz407 31930326

[3] Desouza D D Hodaie M Davis K D. Structural magnetic resonance imaging can identify trigeminal system abnormalities in classical trigeminal neuralgia. Front Neuroanat. (2016) 10:95. 10.3389/fnana.2016.00095 27807409 PMC5070392

[4] Zhao G Sun X Zhang Z Yang H Zheng X Feng B. Clinical efficacy of MVD combined with PSR in the treatment of primary trigeminal neuralgia. Exp Ther Med. (2020) 20:1582–8. 10.3892/etm.2020.8871 32742390 PMC7388245

[5] Willsey MS Collins KL Conrad EC Chubb HA Patil PG. Diffusion tensor imaging reveals microstructural differences between subtypes of trigeminal neuralgia. J Neurosurg. (2019) 19:1–7. 10.3171/2019.4.JNS19299 31323635

[6] Desouza DD Hodaie M Davis KD. Abnormal trigeminal nerve microstructure and brain white matter in idiopathic trigeminal neuralgia. Pain. (2014) 155:37–44. 10.1016/j.pain.2013.08.029 23999058

[7] Chen ST Yang JT Weng HH Wang HL Yeh MY Tsai YH. Diffusion tensor imaging for assessment of microstructural changes associate with treatment outcome at one-year after radiofrequency Rhizotomy in trigeminal neuralgia. BMC Neurol. (2019) 19:62. 10.1186/s12883-019-1295-5 30979362 PMC6460667

[8] Lutz J Thon N Stahl R Lummel N Tonn JC Linn J. Microstructural alterations in trigeminal neuralgia determined by diffusion tensor imaging are independent of symptom duration, severity, and type of neurovascular conflict. J Neurosurg. (2016) 124:823–30. 10.3171/2015.2.JNS142587 26406792

[9] Lutz J Linn J Mehrkens JH Thon N Stahl R Seelos K. Trigeminal neuralgia due to neurovascular compression: high-spatial-resolution diffusion-tensor imaging reveals microstructural neural changes. Radiology. (2011) 258:524–30. 10.1148/radiol.10100477 21062923

[10] Chen F Chen L Li W Li L Xu X Li W. Pre-operative declining proportion of fractional anisotropy of trigeminal nerve is correlated with the outcome of micro-vascular decompression surgery. BMC Neurol. (2016) 16:106. 10.1186/s12883-016-0620-5 27422267 PMC4947245

[11] Wu M Qiu J Jiang X Li M Wang SD Dong Q. Diffusion tensor imaging reveals microstructural alteration of the trigeminal nerve root in classical trigeminal neuralgia without neurovascular compression and correlation with outcome after internal neurolysis. Magn Reson Imaging. (2020) 71:37–44. 10.1016/j.mri.2020.05.006 32439427

[12] Jin Z Bao Y Wang Y Li Z Zheng X Long S. Differences between generalized Q-sampling imaging and diffusion tensor imaging in visualization of crossing neural fibers in the brain. Surg Radiol Anat. (2019) 41:1019–28. 10.1007/s00276-019-02264-1 31144009 PMC6694094

[13] Hodgson K Adluru G Richards LG Majersik JJ Stoddard G Adluru N. Predicting motor outcomes in stroke patients using diffusion spectrum MRI microstructural measures. Front Neurol. (2019) 10:72. 10.3389/fneur.2019.00072 30833925 PMC6387951

[14] Leng B Han S Bao Y Zhang H Wang Y Wu Y. The uncinate fasciculus as observed using diffusion spectrum imaging in the human brain. Neuroradiology. (2016) 58:595–606. 10.1007/s00234-016-1650-9 26906111

[15] Basser PJ. Inferring microstructural features and the physiological state of tissues from diffusion-weighted images. NMR Biomed. (1995) 8:333–44. 10.1002/nbm.1940080707 8739270

[16] Fritzsche KH Laun FB Meinzer HP Stieltjes B. Opportunities and pitfalls in the quantification of fiber integrity: what can we gain from Q-ball imaging? Neuroimage. (2010) 51:242–51. 10.1016/j.neuroimage.2010.02.007 20149879

[17] Yeh FC Wedeen VJ Tseng WY. Generalized q-sampling imaging. IEEE Trans Med Imaging. (2010) 29:1626–35. 10.1109/TMI.2010.2045126 20304721

[18] Yeh FC Liu L Hitchens TK Wu YL. Mapping immune cell infiltration using restricted diffusion MRI. Magn Reson Med. (2017) 77:603–12. 10.1002/mrm.26143 26843524 PMC8052951

[19] Lin HY Perry A Cocchi L Roberts JA Tseng WI Breakspear M. Development of frontoparietal connectivity predicts longitudinal symptom changes in young people with autism spectrum disorder. Transl Psychiatry. (2019) 9:86. 10.1038/s41398-019-0418-5 30755585 PMC6372645

[20] Wu CH Hwang TJ Chen PJ Chou TL Hsu YC Liu CM. Reduced structural integrity and functional lateralization of the dorsal language pathway correlate with hallucinations in schizophrenia: a combined diffusion spectrum imaging and functional magnetic resonance imaging study. Psychiatry Res. (2014) 224:303–10. 10.1016/j.pscychresns.2014.08.010 25241043

[21] Lin YC Shih YC Tseng WY Chu YH Wu MT Chen TF. Cingulum correlates of cognitive functions in patients with mild cognitive impairment and early Alzheimer's disease: a diffusion spectrum imaging study. Brain Topogr. (2014) 27:393–402. 10.1007/s10548-013-0346-2 24414091

[22] Romascano D Meskaldji DE Bonnier G Simioni S Rotzinger D Lin YC. Multicontrast connectometry: a new tool to assess cerebellum alterations in early relapsing-remitting multiple sclerosis. Hum Brain Mapp. (2015) 36:1609–19. 10.1002/hbm.22698 25421928 PMC6869568

[23] Zhang Y Zhang Z Jia X Guan X Lyu Y Yang J. Imaging parameters of the ipsilateral medial geniculate body may predict prognosis of patients with idiopathic unilateral sudden sensorineural hearing loss on the basis of diffusion spectrum imaging. AJNR Am J Neuroradiol. (2021) 42:152–9. 10.3174/ajnr.A6874 33214182 PMC7814812

[24] Yoshino M Abhinav K Yeh FC Panesar S Fernandes D Pathak S. Visualization of cranial nerves using high-definition fiber tractography. Neurosurgery. (2016) 79:146–65. 10.1227/neu.0000000000001241 27070917

[25] Yeh FC Verstynen TD Wang Y Fernández-Miranda JC Tseng WY. Deterministic diffusion fiber tracking improved by quantitative anisotropy. PLoS ONE. (2013) 8:e80713. 10.1371/journal.pone.0080713 24348913 PMC3858183

[26] Chai W You C Zhang W Peng W Tan L Guan Y. Diffusion tensor imaging of microstructural alterations in the trigeminal nerve due to neurovascular contact/compression. Acta Neurochir (Wien). (2019) 161:1407–13. 10.1007/s00701-019-03851-2 31065894

[27] Moon HC You ST Baek HM Jeon YJ Park CA Cheong JJ. 7.0 Tesla MRI tractography in patients with trigeminal neuralgia. Magn Reson Imaging. (2018) 54:265–70. 10.1016/j.mri.2017.12.033 29305127

[28] Wedeen VJ Wang RP Schmahmann JD Benner T Tseng WY Dai G. Diffusion spectrum magnetic resonance imaging (DSI) tractography of crossing fibers. Neuroimage. (2008) 41:1267–77. 10.1016/j.neuroimage.2008.03.036 18495497

[29] Bao Y Wang Y Wang W Wang Y. The superior fronto-occipital fasciculus in the human brain revealed by diffusion spectrum imaging tractography: an anatomical reality or a methodological artifact? Front Neuroanat. (2017) 11:119. 10.3389/fnana.2017.00119 29321729 PMC5733543

[30] Wu Y Sun D Wang Y Wang Y Wang Y. Tracing short connections of the temporo-parieto-occipital region in the human brain using diffusion spectrum imaging and fiber dissection. Brain Res. (2016) 1646:152–9. 10.1016/j.brainres.2016.05.046 27235864

[31] Tuch DS. Q-ball imaging. Magn Reson Med. (2004) 52:1358–72. 10.1002/mrm.20279 15562495

[32] Holliday KL Mcbeth J. Recent advances in the understanding of genetic susceptibility to chronic pain and somatic symptoms. Curr Rheumatol Rep. (2011) 13:521–7. 10.1007/s11926-011-0208-4 21877183

[33] Apfelbaum RI. Neurovascular decompression: the procedure of choice? Clin Neurosurg. (2000) 46:473–98. 10944696

[34] Li TY Chen VC Yeh DC Huang SL Chen CN Chai JW. Investigation of chemotherapy-induced brain structural alterations in breast cancer patients with generalized q-sampling MRI and graph theoretical analysis. BMC Cancer. (2018) 18:1211. 10.1186/s12885-018-5113-z 30514266 PMC6280365

